# Diagnostic and Therapeutic Challenges in Multifocal MRSA Pyomyositis: A Case Report

**DOI:** 10.1155/crcc/1360623

**Published:** 2025-07-31

**Authors:** Tommaso Foggi Viligiardi, Lucia Negro, Alessandro Coppa, Simone Cipani

**Affiliations:** ^1^School of Specialization in Anesthesia, Resuscitation, Intensive Care, and Pain Medicine, Università degli Studi di Firenze, Florence, Italy; ^2^Department of Emergency and Urgent Care, Azienda Ospedaliero-Universitaria Careggi, Florence, Italy; ^3^Department of Emergency and Urgent Care, Azienda USL Toscana Centro, Florence, Italy

**Keywords:** abscess drainage, critical care, immunocompromised host, MRSA, pyomyositis, septic emboli, septic shock

## Abstract

**Background:** Pyomyositis is a rare bacterial infection of skeletal muscle, historically associated with tropical climates but increasingly observed in temperate areas, particularly among immunocompromised individuals. Its nonspecific early symptoms often overlap with other soft-tissue infections, causing diagnostic delay.

**Case Presentation:** We report the case of a 64-year-old lumberjack with poorly controlled diabetes and a remote history of lymphoma, who developed multifocal MRSA pyomyositis with septic pulmonary emboli following orthopedic trauma and surgery. The condition rapidly evolved into septic shock requiring ICU admission, targeted antibiotic therapy, and multiple image-guided drainages. The patient progressively recovered and was discharged to rehabilitation on Postoperative Day 45.

**Conclusion:**Pyomyositis should be considered in immunocompromised patients presenting with localized pain, systemic symptoms, and recent trauma. Early imaging, microbiological confirmation, and multidisciplinary management are critical for improving outcomes.

## 1. Introduction

Pyomyositis is a primary, purulent bacterial infection of skeletal muscle, most commonly caused by *Staphylococcus aureus*, including methicillin-resistant strains (methicillin-resistant *Staphylococcus aureus* [MRSA]) [1]. Once regarded as a disease confined to tropical climates, pyomyositis has emerged as a growing concern in temperate regions, particularly among immunocompromised populations such as individuals with diabetes mellitus, malignancies, HIV infection, or those receiving immunosuppressive therapies [2–4].

Clinically, the disease progresses through three distinct stages: an initial invasive phase characterized by localized muscle pain, low-grade fever, and minimal systemic symptoms; a suppurative phase marked by abscess formation and systemic inflammatory signs; and a septic phase, which may involve bacteremia, septic shock, and distant metastatic infections [1, 5]. Because its clinical presentation often overlaps with other soft tissue infections such as cellulitis, osteomyelitis, or necrotizing fasciitis, diagnostic delays are frequent and potentially detrimental [6, 7].

Magnetic resonance imaging (MRI) is the gold standard for early detection and accurate delineation of muscular involvement [6]. Definitive diagnosis hinges on microbiological confirmation, typically through aspirates obtained from abscess cavities or positive blood cultures [7]. Management necessitates prompt initiation of empiric intravenous antibiotics targeting MRSA and Gram-negative organisms, followed by pathogen-specific adjustments based on culture sensitivities. Equally crucial is the timely drainage of abscesses, via surgical intervention or image-guided aspiration [8, 9].

Early recognition and intervention are imperative to mitigate progression to systemic complications, including sepsis and multiorgan dysfunction, and to optimize clinical outcomes [4, 9].

## 2. Case Presentation

A 64-year-old male lumberjack presented to the emergency department after falling down 18 steps. He reported lumbar and right elbow pain. His past medical history included

• Type 2 diabetes mellitus, poorly controlled with oral agents and insulin;
• obesity (BMI 31);
• remote history of lymphoma treated with chemotherapy and bone marrow transplantation approximately 30 years ago, with sustained complete remission and no evidence of disease recurrence to date;
• right total hip arthroplasty (2 years before);
• right rotator cuff repair (2 months prior).

**Figure 1:**
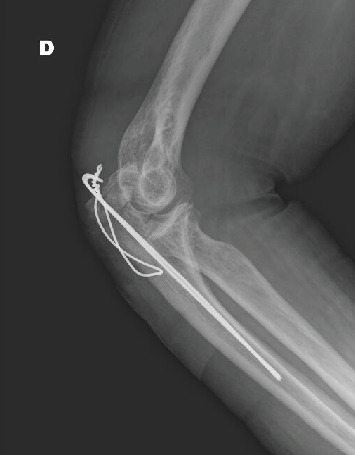
X-ray of the stabilization of the fracture of the olecranon with *Zuggurtung* technique.

Radiologic evaluation showed a comminuted fracture of the right olecranon and a displaced fracture of the right ischiopubic ramus. The patient underwent elbow fixation with the *Zuggurtung* technique under regional anesthesia (Figure 1) without perioperative complications. He was discharged on Postoperative Day (POD) 6 in good general condition.

On POD 10, he returned to the emergency department with fever (38.7°C), altered mental status, and dyspnea. His operated limb was swollen, warm, and exuding purulent material. Arterial blood gas revealed hypoxemia (PaO₂ 57 mmHg), respiratory alkalosis, and elevated lactate (5.4 mmol/L). Labs showed CRP 91.6 mg/L, procalcitonin 6.2 ng/mL, and severe thrombocytopenia (23,000/*μ*L).

**Figure 2:**
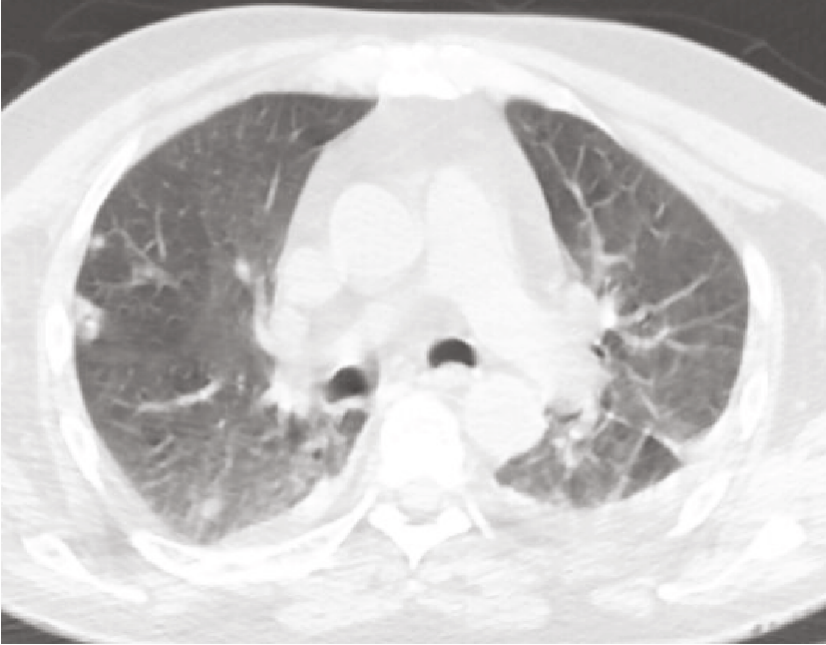
Chest CT scan with multiple irregularly nodular formations.

Empiric antibiotic therapy with ceftriaxone was initiated upon the patient's arrival to the ED. A chest CT (Figure 2) identified bilateral nodular opacities and right lower lobe consolidation, suggestive of septic pulmonary emboli.

Within hours, his clinical condition rapidly worsened with escalating signs of septic shock, including hypotension, tachypnea, and altered mental status. He was urgently transferred to the intensive care unit, where he required endotracheal intubation, mechanical ventilation, and vasopressor support. Given the severity of the presentation, antimicrobial therapy was broadened to meropenem, fosfomycin, and daptomycin to ensure adequate coverage for resistant pathogens. Microbiological cultures from blood, bronchoalveolar lavage, and wound samples subsequently confirmed the presence of MRSA. Based on these findings, the regimen was rationalized to daptomycin and linezolid. As severe thrombocytopenia persisted and to minimize the risk of further hematologic compromise, linezolid was later replaced with ceftobiprole.

**Figure 3:**
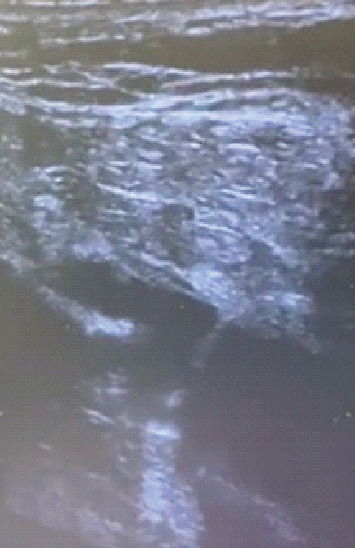
Ultrasound-guided drainage of the right shoulder's abscess.

**Figure 4:**
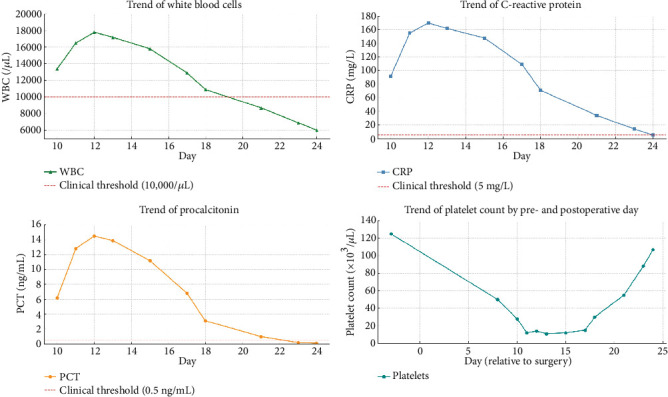
Trends in inflammatory and hematologic markers during postoperative hospitalization. Graphs illustrate the daily evolution of white blood cell count (WBC), C-reactive protein (CRP), procalcitonin (PCT), and platelet count in relation to pre- and postoperative days.

Transesophageal echocardiography excluded infective endocarditis. Soft tissue ultrasound and CT showed intramuscular abscesses involving the right forearm, shoulder, hip, and bilateral psoas muscles. On POD 18, ultrasound-guided drainage of the right shoulder yielded purulent MRSA-positive material (Figure 3). Over subsequent days, inflammatory markers normalized, allowing tracheostomy and ventilator weaning (Figure 4).

**Figure 5:**
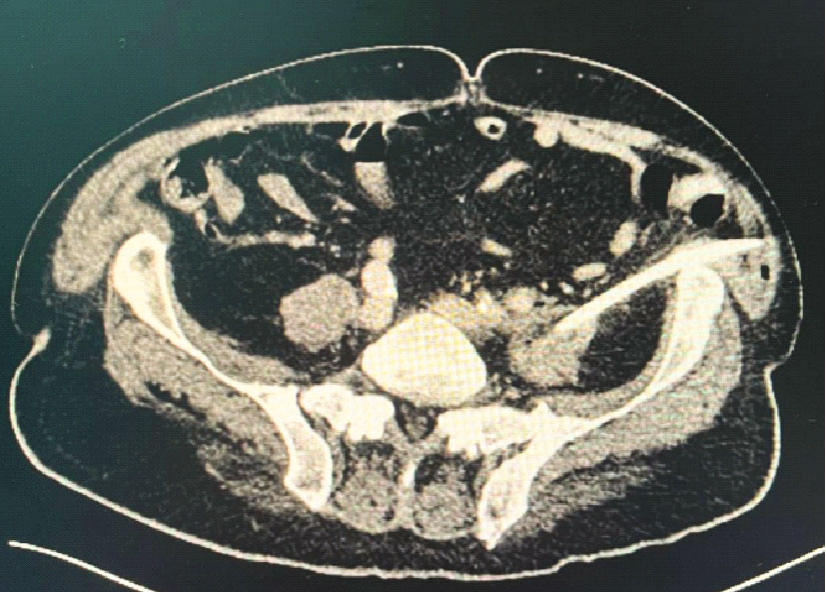
CT-guided retroperitoneal drainage of the psoas' abscess.

On POD 35, spinal MRI confirmed multiple collections in the psoas and paraspinal muscles. CT-guided retroperitoneal drainage was performed (Figure 5). With sustained clinical improvement and negative inflammation markers, antibiotics were discontinued on POD 43.

The patient was discharged to rehabilitation on POD 45.

## 3. Discussion

This case illustrates the complexities of diagnosing pyomyositis in nonendemic settings. In our patient, early symptoms were subtle and mimicked a postoperative infection. Only after systemic deterioration and imaging identification of multifocal abscesses and septic pulmonary emboli was the diagnosis of MRSA pyomyositis established.

Risk factors in this case included poorly controlled diabetes, remote chemotherapy-induced immunosuppression, recent surgery, and possible microtrauma from the patient's occupation. The absence of endocarditis and the presence of multiple muscle abscesses supported the diagnosis of hematogenous MRSA pyomyositis.

**Figure 6:**
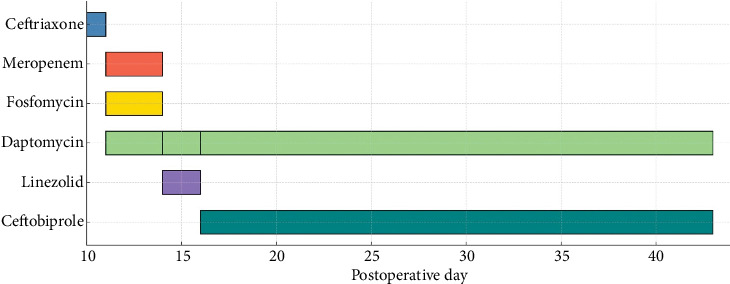
Timeline of antimicrobial therapy administered during hospitalization.

Given the severity of the infection and the evolving clinical picture, the patient required a complex, multistep antimicrobial strategy that was gradually refined based on microbiological data and clinical response (Figure 6).

Therapy was discontinued on POD 43 after full resolution of systemic signs.

Early multidisciplinary management, including ICU support, targeted antibiotics, and percutaneous drainages, was essential for recovery. This case highlights the importance of considering pyomyositis even in temperate climates when risk factors and systemic signs are present.

## 4. Conclusion

Pyomyositis should be included in the differential diagnosis of immunocompromised patients with unexplained muscular pain and systemic symptoms, especially after trauma or surgery. Early recognition, imaging, microbiological confirmation, and aggressive source control can significantly reduce complications and improve outcomes.

This case reflects known predisposing factors such as diabetes and recent trauma, but is notable for its multifocal muscle involvement, septic pulmonary emboli in the absence of endocarditis, and prolonged intensive care course.

The occurrence of such extensive MRSA pyomyositis in a patient with remote resolved lymphoma is exceptionally uncommon. It broadens the current spectrum of disease presentation and reinforces the importance of early recognition and aggressive targeted management in complex hosts to optimize recovery.

## Data Availability

All data generated or analyzed during this study are included in this published article.
